# Availability of Authorizations from EMA and FDA for Age-Appropriate Medicines Contained in the WHO Essential Medicines List for Children 2019

**DOI:** 10.3390/pharmaceutics12040316

**Published:** 2020-04-01

**Authors:** Jose-Manuel delMoral-Sanchez, Isabel Gonzalez-Alvarez, Marta Gonzalez-Alvarez, Andres Navarro-Ruiz, Marival Bermejo

**Affiliations:** 1 Department of Pharmacokinetics and Pharmaceutical Technology, Miguel Hernandez University, 03550 San Juan de Alicante, Spain; jmoral@umh.es (J.-M.d.-S.); marta.gonzalez@umh.es (M.G.-A.); mbermejo@umh.es (M.B.); 2 Institute of Molecular and Cellular Biology of Miguel Hernandez University, 03202 Elche, Spain; 3 Pharmacy Service, General University Hospital of Elche, 03203 Elche, Spain; navarro_and@gva.es

**Keywords:** commercial availability, pediatrics, age appropriate, pharmaceutical preparations, safety, regulatory

## Abstract

Lack of age-appropriate commercially drug products availability is a common problem in pediatric therapeutics; this population needs improved and safer drug delivery. In addition, biopharmaceutic aspects, dosage requirements, and swallowing abilities demand pediatric forms different to adult formulations. The objective of this study was to evaluate the authorization availability from United States Food and Drug Administration (FDA) and European Medicines Agency (EMA) of oral essential medicines for children and analyze its age-appropriateness for oral administration in children. All oral drugs from 7th List of Essential Medicines for Children by World Health Organization (WHO) were selected. Availability of commercial drug products was collected from OrangeBook, Spanish drug product catalogue, British electronic Medicines Compendium, and the International Vademecum. Tablets, effervescent tablets, and capsules were considered as not age-appropriate forms. Liquid forms, powder for oral suspension, mini tablets, granules, and soluble films were considered as age-appropriate forms due to their flexibility. More than 80% of the studied drugs possess a commercial authorization in oral forms in both EMA and FDA. Nevertheless, around 50% of these formulations are not age-appropriate for most pediatric groups. This study shows the lack of age-appropriate medicines for children. More efforts are needed to improve development and approval of pediatric medicines.

## 1. Introduction

Nowadays, formulation research and development in the pediatric area remains essential and is required [1,2]. Governments allocate considerable efforts to promote the availability of age-appropriate, safe, and effective pediatric medicines by regulatory incentives [3,4]. Most drugs are often not appropriate for pediatrics due to the drug dosage form (tablets or capsules) and strengths. A lack of medicines specifically developed for children may be managed by preparing medicines extemporaneously or by manipulating dosage forms designed for adults, e.g., splitting tablets, crushing, and administering with food or liquid. Children cannot be considered small adults because of pharmacokinetic, pharmacodynamics, physiological, and anatomical differences.

Physiological aspects like the pH of the gastrointestinal tract [5,6] or expression of drug-metabolizing enzymes and transporters [7] are major facts for oral drug absorption and can alter the bioavailability of the administered drug [8]. These facts change with age and appear to be necessary to develop age-appropriate formulations. In respect with drug administration, children have different capabilities from adults, such as palatability or swallowing facility.

Marketed formulations are intended for adults predominantly, so pharmaceutical compounding and manipulations are often necessary, such as crushing tablets, dispersing content capsule or crushed tablets in liquid excipients, or using parenteral formulation for oral administration [9,10].

In connection with appropriate drug dosage forms for oral administration, the most suitable forms are syrups, oral solutions, or suspensions. They ensure the administration of different doses with a single medicine and the possibility of mixing them with food or beverages [11,12].

Nowadays, new solid oral formulations are emerging [13]. These novel pediatric specific formulations include: Mini tablets, oral-soluble films, oral powders and granulates, orally disintegrating tablets, chewable tablets, scored tablets, and sprinkle capsules [14]. New solid oral forms could be age appropriate if the flexibility of dosing can be achieved and may avoid the use of harmful excipients and palatability issues for liquid medicines.

Apart from the lack of age-appropriate medicines, there is almost a total absence of medicines approved for neonates and common formulations are used because there are no suitable alternatives [4].

World Health Organization (WHO) Essential Medicines List for children (EMLc) considers the efficacious, cost-effective, and safest drugs for priority pediatric pathologies for a basic healthcare system [15]. Listed drugs with immediate release oral forms from 7th Edition (2019) of EMLc were selected to examine the authorization availability of age-appropriate medicines from the European Medicines Agency (EMA) and United States Food and Drug Administration (FDA).

## 2. Materials and Methods

All drugs with an available oral administration route from 7th List of Essential Medicines for Children [15] were selected.

Drug products’ information from FDA was collected in July of 2019 from OrangeBook [16]. Information from EMA was listed from Spanish drug product catalogue (CIMA) [17], British electronic Medicines Compendium (eMC) [18], and the International Vademecum [19] due to these databases being public access formularies. International Vademecum was selected because it is a database with data from the member countries of EMA.

Selected drugs were classified into orally available if the drug is commercially available in any drug dosage form of oral administration.

Solid dosage forms like tablets, effervescent tablets, or capsules were considered as not age-appropriate forms for pediatrics due to their poor versatility for dosage administration. In the present study, pediatric age range was considered as 0 to 12 years because it is the contemplated age range in EMLc.

Drug dosage forms like syrups, oral solutions or suspensions, chewable tablets for pediatrics, granules, oral-soluble films, minitablets, or powder for oral suspension were assigned to age-appropriate forms for pediatric population. Furthermore, the first authorization year in FDA and EMA of age-appropriate forms was included.

Additionally, selected drugs were classified into WHO Anatomical Therapeutic Chemical (ATC) classification system (first level—anatomical main group), the recommended system by WHO for international drug utilization studies [20,21], and an intragroup analysis was done about drugs with age-appropriate forms.

The percentages of several analyses were the result of the following equations:

% availability = (orally commercial drugs available)/(selected drugs),(1)

% age-appropriate = (drugs with oral pediatric age-appropriate form)/(orally commercial drugs available),(2)

% age-appropriate (per therapeutic group) = (drugs with oral pediatric age-appropriate form from therapeutic group x)/(drugs within therapeutic group x).(3)

## 3. Results

### 3.1. Availability and Suitability of Oral Formulations

Table A1 shows all 149 drugs included as essential medicines in children for oral administration and its classification as commercially available or age-appropriate oral formulation for pediatrics in EMA or FDA. Additionally, new solid oral forms and age-appropriate forms that emerged in order to meet requirements years after their first authorization are highlighted.

**Figure 1:**
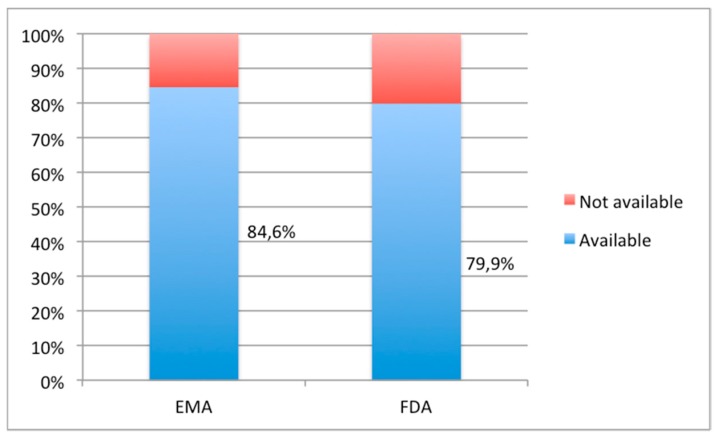
Percentage of oral dosage forms in Essential Medicines List for children (EMLc) 2019 listed as authorized medicines by European Medicines Agency (EMA) and United States Food and Drug Administration (FDA).

**Figure 2:**
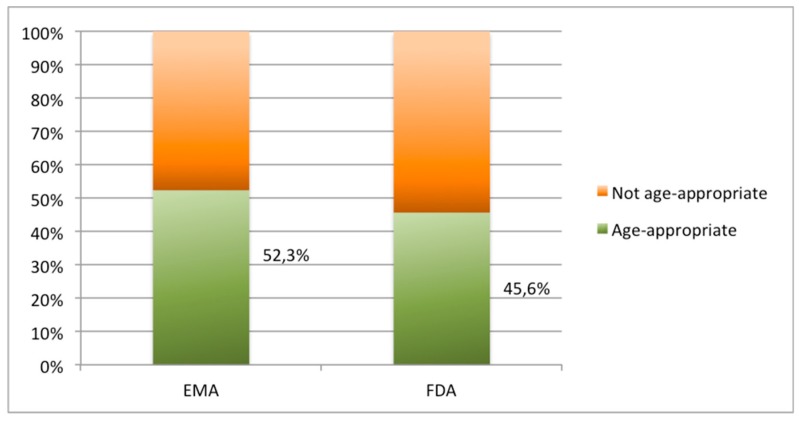
Percentage of oral dosage forms in EMLc 2019 listed as authorized age-appropriate medicines by EMA and FDA.

Figure 1 shows the authorization availability of orally commercial forms both in EMA and FDA. Figure 2 illustrates the age appropriateness of the orally commercial formulations both in EMA and FDA.

### 3.2. Therapeutic Class Distribution

**Figure 3:**
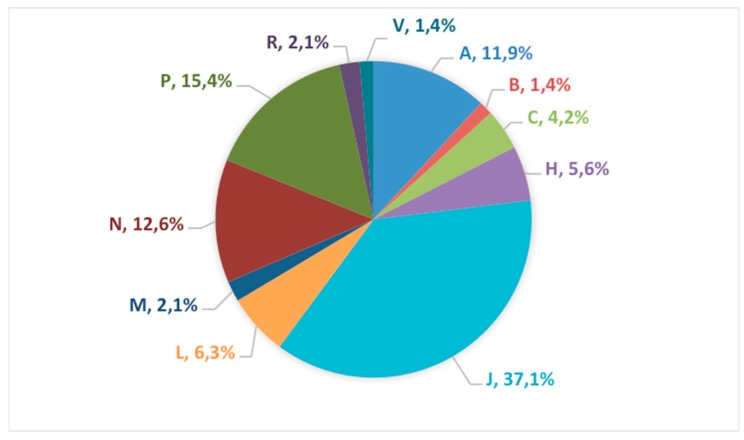
Percentage of oral drugs listed in EMLc by Anatomical Therapeutic Chemical (ATC).

Figure 3 shows the therapeutic class distribution by the ATC Classification System regarding the analysis per groups, Therapeutic classes to which the selected oral drugs belong were: A: Alimentary tract and metabolism; B: Blood and blood forming organs; C: Cardiovascular system; H: Systemic Hormonal preparations; J: Anti-infective for systemic use; L: Antineoplastic and immunomodulation agents; M: Muscular-skeletal system; N: Nervous system; P: Antiparasitic products; R: Respiratory system; and V: Various.

**Figure 4:**
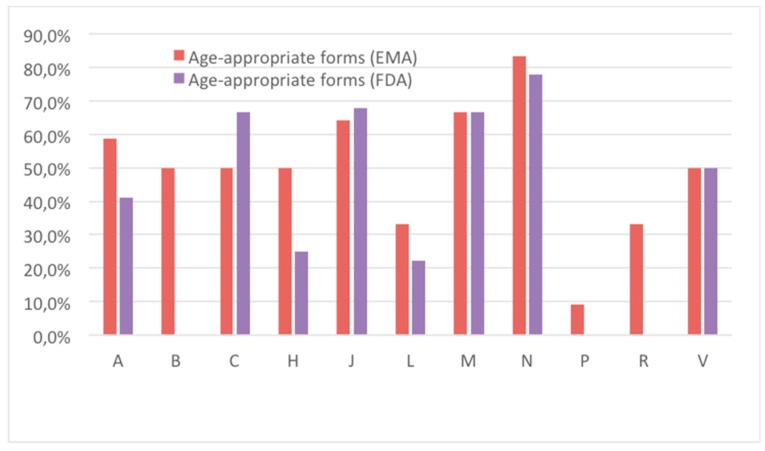
Comparison of the availability of authorized oral age-appropriate dosage forms within therapeutic (ATC) classes.

Figure 4 shows the percentage of the age appropriateness of available drugs with respect to ATC classes.

## 4. Discussion

Specific development of drug products for pediatrics has been inappropriate because of the lower prevalence of diseases in children in comparison with adults, and also relates to commercial reward. The scarcity of resources with regard to pediatric pharmacotherapy is an acknowledged gap by worldwide governments and regulatory agencies [22]. The first step was taken in 1970s when FDA stated that most prescription drugs were administered empirically and it called for innovative programs to provide pediatric information [23] and recognized that excluding children from clinical trials was an unethical fact and could create risk situations [4]. Since then, many useful efforts have been devoted to promoting research in pediatric pharmacotherapy. Even now, more than 40 years later, age-appropriate medicines for children are still not available. In the present study, only drugs from the List of Essential Medicines for Children of WHO were analyzed, but there are considerably more drugs to analyze that are also not available and are used in off-label conditions.

The commercial availability of oral forms of selected drugs proved to be high (84.6% in EMA vs. 79.9% in FDA) (Figure 1).

Differences in the formularies or catalogues of all countries that comprise EMA were an identified limitation. The presence of a drug in one of the drug catalogues does not mean that the product is in fact available in all EMA countries. An additional limitation was not being able to access all the official catalogues of the member countries.

Another limitation was that the essential list by WHO is made for worldwide countries, so all drugs listed are perhaps not relevant for all clinical settings. For example, fexinidazole is an antiparasitic drug indicated for African Chagas without authorization available in the FDA nor in EMA (Table A1). There are several factors that determine the availability of therapies in a region or market, including disease prevalence and an available patient population to complete development.

Highlighted drug forms (Table A1), such chewable tablets (i.e., Ibuprofen or Lamotrigine), oral-soluble films (i.e., Ondansetron), or prolonged release granules (i.e., Valproic acid), were found as authorized medicines with age-appropriate and innovative forms. Other updated formulations of drugs, such as Propranolol (oral solution 4mg/mL) and Methotrexate (oral solution 2 mg/mL), were found and they were developed to meet special pediatric requirements and pathologies (propranolol for infantile hemangioma and Methotrexate for acute lymphoblastic leukemia and polyarticular juvenile idiopathic arthritis). It should be noted that these new formulations were developed and authorized from 2007, when the EMA legislation took effect and the FDA was more effective, with the formation of the Pediatric Review Committee.

In regard to the ATC class distribution (Figure 3), anti-infective drugs of J class (anti-infective for systemic use) P class (antiparasitic products) constitute the largest group (more than 50%).

It is noteworthy the an extremely lower percentage of age appropriateness of antiparasitic formulations is one of the majority groups (Figure 4). Groups B (blood), P (antiparasitic products), and R (respiratory system) are the ones in the FDA area showing a lack of age-appropriate formulations. Conversely, N class (nervous system) drugs had the highest percentage of age-appropriate forms in both EMA and FDA, followed by J (anti-infective for systemic use) and M (muscular-skeletal system) groups.

Furthermore, the presence of drugs used in the treatment of neglected diseases (21.7% of included drugs) should be noted. While in most cases these are commercially available, very few are age-appropriate dosage forms for pediatric oral administration, as well as posological adjustment. Frequently, formulations are compounded from adult drug products to avoid the problem, but as explained above, this practice is not considered advisable but unavoidable, and can lead to biopharmaceutical and safety problems [12,24].

Despite the high accessibility of oral drug products, a gap can be identified regarding the suitability of drug dosage forms to pediatrics (52.3% in EMA vs. 45.6% in FDA) (Figure 2). Pediatric populations need maximal dosing flexibility, palatability, and safety [25,26].

Regulatory authorities are aware of the lack of age-appropriate forms and the need to use extemporaneous formulations from active product ingredients or even from manipulation of adult dosage forms as it was recognized in the Reflection Paper: Formulations of choice for the pediatric population by EMEA in 2006 [12]. A potential risk for the consistent performance of those compounded formulations is the effect of excipients on the release, transit, and absorption of low solubility and/or low permeability drugs. The selection of suitable excipients and its age-related safety profiles are especially critical in drug product development and pharmaceutical compounding intended for neonates and young children [27].

Another potential risk, associated with compounding, is the solution osmolarity in liquid formulations, which eventually may affect the membrane permeation rate [28]. Any change on absorption could be in particular problematic in drugs with a narrow therapeutic range [25]. The recognition nowadays that excipients could not be “inert” components but have an effect of gastrointestinal motility, permeability, or fluid balance [26,29] is of especial relevance in children due to its rapid developmental changes in intestine physiology and because our knowledge at this level is still scarce [29].

The Biopharmaceutics Classification System (BCS) is a widely evolved and used tool in the development of medicines in adults [30] as well as physiologically based pharmacokinetics (PBPK) modelling and the dissolution test (in vitro) [31]. The development of a pediatric biopharmaceutics classification system (pBCS) could help to identify those drugs for which harmonization of compounded formulas would be advisable [6,28,29] as their absorption rate/extent is particularly sensitive to the effect of excipients on drug solubility, permeability, or the dissolution rate.

## 5. Conclusions

This quantitative evaluation confirms the need for improvements in drug delivery in pediatrics and the lack of age-appropriate medicines in many therapeutic areas. Currently, real efforts are being made to improve the development and approval of drug products aimed for children because of global requirements. These formulations must be able to adapt to pediatric oral biopharmaceutics and capabilities.

Although it is a difficult task to carry out, this paper calls for suitable pediatric formulations that can be orally administered in an appropriate form based on dose flexibility, swallowability, and palatability. In addition, thinking regarding older products and an attempt to develop age-appropriate medicines, as with the drugs that have already been achieved by new oral solid formulations, should be prioritized.

## Appendix A

**Table A1:** Authorization availability and pediatric oral age-appropriate forms of EMLc 2019 by WHO. Oral dosage forms in italics are those that are not age appropriate for oral administration. The authorization year of age-appropriate medicines is indicated in parenthesis. Innovative forms and other age-appropriate forms that emerged in order to meet requirements years after their first authorization are shown as underlined. Age-appr: age-appropriate.

Drug	European Medicines Agency			Food and Drug Administration		
	Oral Availability	Age-appr	Formulation	Oral Availability	Age-appr	Formulation
Abacavir	yes	yes	oral solution 20 mg/mL (1999)	yes	yes	oral solution 20 mg/mL (1998)
Acetylcysteine	yes	yes	oral solution 20 mg/mL (2000) powder for oral suspension 100, 200 mg (2000) *effervescent tablet 600 mg*	yes	yes	oral solution 10% and 20% (1989) *effervescent tablet 500 mg and 2500 mg*
Acetylsalicylic acid	yes	no	*tablet 100, 150, 300, 500 mg*	yes	no	*tablet 500 mg* *capsule 162.5, 325 mg*
Acyclovir	yes	yes	oral suspension 80 mg/mL (1992) *tablet 200–800 mg*	yes	yes	oral suspension 200 mg/5 mL (1989) *tablet 50–800 mg*
Albendazole	yes	no	*tablet 400 mg*	yes	no	*tablet 200 mg*
Allopurinol	yes	no	*tablet 100, 300 mg*	yes	no	*tablet 100, 300 mg*
Amitriptyline	yes	yes	oral solution 50 mg/mL, 10 mg/mL (2010) *tablet 25–150 mg*	yes	no	*tablet 25* *–150 mg*
Amodiaquine	no	no	*-*	no	no	-
Amoxicillin *(*in association with clavulanic acid see clavulanic acid)*	yes	yes	oral suspension 250 mg/5 mL (1974) powder for oral suspension 500 mg (1990) *tablet 500 mg*	yes	yes	oral solution 250 mg/mL and 400 mg/mL (1982) chewable tablet 125, 250 mg (1992) *tablet 250, 500 mg*
Artesunate	no	no	*-*	no	no	Artemether is available
Ascorbic acid	yes	no	*tablet 50, 100, 200 mg*	no	no	-
Atazanavir	yes	yes	powder for oral suspension 50 mg (2016) *capsule 150, 200, 300 mg*	yes	yes	powder for oral suspension 50 mg (2014) *capsule 150, 200, 300 mg*
Azathioprine	yes	no	*tablet 50 mg*	yes	no	*tablet 50 mg*
Azithromycin	yes	yes	oral suspension 200 mg/5 mL (1992) powder for oral suspension 500 mg (1992) *tablet 500 mg*	yes	yes	oral suspension 200 mg/5 mL (2006) *tablet 500 mg*
Benznidazole	no	no	*-*	no	no	-
Caffeine citrate	yes	yes	oral solution 10 mg/mL (2018)	yes	yes	oral solution 10 mg/mL (2000)
Calcium folinate	yes	yes	oral suspension 1 mg/12 mL (1966)	no	no	-
Calcium gluconate	yes	no	*effervescent tablet 1000 mg*	no	no	-
Carbamazepine	yes	no	*tablet 200, 400 mg*	yes	yes	oral suspension 100 mg/5 mL (1987) *capsule 200 mg*
Cefixime	yes	yes	oral suspension 100 mg/5 mL (1993) *capsule 200, 400 mg*	yes	yes	oral suspension 100 mg/5 mL, 200 mg/5 mL (2007) *chewable tablet 100,150, 200 mg*
Cephalexin	yes	yes	oral suspension 250 mg/5 mL, 125 mg/5 mL (1996) *capsule 500 mg*	yes	yes	oral suspension 125 mg/5 mL, 250 mg/5 mL (1987) *capsule 750 mg*
Chloramphenicol	no	no	-	no	no	-
Chloroquine	yes	no	*tablet 250 mg*	yes	no	*tablet 150, 300 mg*
Chlorpromazine	yes	yes	oral solution 40 mg/mL (1955) *tablet 25, 100 mg*	yes	no	*tablet 10* *–200 mg*
Cholecalciferol	yes	yes	oral solution 2000 UI/mL (1955)	no	no	-
Ciprofloxacin	yes	yes	oral suspension 100 mg/mL (1999) *tablet 250–750 mg*	yes	yes	oral suspension 250 mg/5 mL, 500 mg/5 mL (1997) *tablet 100–750 mg*
Clarithromycin	yes	yes	oral suspension 125 mg/5 mL, 200 mg/5 mL (2004) *tablet 250, 500 mg*	yes	yes	oral suspension 125 mg/5 mL, 200 mg/5 mL (2002) *tablet 250, 500 mg*
Clavulanic acid	yes	yes	oral suspension 100/12.5 mg/mL (1991) powder for oral suspension 500/125 mg and 875/125 mg (1982) *tablet 500/125 mg and 875/125 mg*	yes	yes	oral suspension (multiple doses) (1984) chewable tablet 200/28.5 mg, 400/57 mg (2005) *tablet (250–875 mg/125 mg/mL)*
Clindamycin	yes	no	*capsule 150, 300 mg*	yes	yes	oral solution 75 mg/5 mL (1986) *capsule 150, 300 mg*
Clofazimine	yes	no	*capsule 50 mg*	yes	no	*capsule 50 mg*
Cloxacillin	yes	yes	oral suspension 125 mg/5 mL (1965) *capsule 500 mg*	yes	no	*capsule 125, 250, 500 mg*
Cyclizine	yes	no	*tablet 50 mg*	no	no	-
Cyclophosphamide	yes	no	*dragee 50 mg*	yes	no	*capsule 25, 50 mg* *tablet 25, 50 mg*
Cycloserine	yes	no	*capsule 250 mg*	yes	no	*capsule 250 mg*
Cyclosporin A	yes	yes	oral solution 100 mg/mL (1983) *capsule 25–100 mg*	yes	yes	oral solution 100 mg/mL (1983) *capsule 25–100 mg*
Dapsone	yes	no	*tablet 50, 100 mg*	yes	no	*tablet 25, 100 mg*
Darunavir	yes	yes	oral suspension 100 mg/mL (2007) *tablet 75–800 mg*	yes	yes	oral suspension 100 mg/mL (2011) *tablet 75–800 mg*
Dasatinib	yes	yes	powder for oral suspension 10 mg/mL (2006) *tablet 20–140 mg*	yes	no	*tablet 20* *–70 mg*
Delamanid	yes	no	*tablet 50 mg*	no	no	-
Dexamethasone	yes	yes	oral solution 10 mg/5 mL, 20 mg/5 mL (2013) *tablet 1, 4, 8 mg*	yes	yes	oral solution 1 mg/mL, 0.5 mg/5 mL (1983) *tablet 0.5–6 mg*
Diazepam	yes	yes	oral solution 2 mg/mL (1965) *tablet 2–25 mg*	yes	yes	oral solution 1 mg/mL, 5 mg/mL (1987) *tablet 2, 5, 10 mg*
Diethylcarbamazine	no	no	*tablet 100 mg*	no	no	-
Digoxin	yes	yes	oral solution 0.05 mg/mL (1960) *tablet 0.25 mg*	yes	yes	oral elixir 0.05 mg/mL (2004) *tablet 0.0625–0.25 mg*
Diloxamide	no	no	-	no	no	-
Docusate sodium	yes	no	*capsule 100 mg*	no	no	-
Dolutegravir	no	no	*tablet 10–50 mg*	no	no	*tablet 10–50 mg*
Doxycycline	yes	yes	oral suspension 50 mg/5 mL (1968) *tablet 100 mg* *capsule 100, 200 mg*	yes	yes	oral suspension 25 mg/5 mL (before 1982) *tablet 50–150 mg,* *capsule 50–100 mg*
Efavirenz	yes	no	*tablet 600 mg* *capsule 50* *–200 mg*	yes	no	*tablet 600 mg* *capsule 50* *–200 mg*
Enalapril	no	no	*tablet 2.5* *–20 mg*	yes	yes	oral solution 1 mg/mL (2013) *tablet 2.5–20 mg*
Entecavir	yes	yes	oral solution 0.05 mg/mL (2006) *tablet 0.5, 1 mg*	yes	yes	oral solution 0.05 mg/mL (2005) *tablet 0.5, 1 mg*
Ethambutol	yes	no	*dragee 400 mg*	yes	no	*tablet 100, 400 mg*
Ethionamide	no	no	-	yes	no	*tablet 250 mg*
Ethosuximide	yes	yes	syrup 250 mg/5 mL (2007) *capsule 250 mg*	yes	yes	syrup 250 mg/5 mL (2003) *capsule 250 mg*
Etoposide	yes	no	*capsule 50 mg*	yes	no	*capsule 50 mg*
Fexinidazole	no	no	-	no	no	-
Fluconazole	yes	yes	oral suspension 200 mg/5 mL and 40 mg/5 mL, 2 mg/mL (1991) *capsule 50–200 mg*	yes	yes	oral suspension 200 mg/5 mL, 50 mg/5 mL (1993) *tablet 50–200 mg*
Flucytosine	no	no	-	yes	no	*capsule 250, 500 mg*
Fludrocortisone	yes	no	*tablet 0.1mg*	yes	no	*tablet 0.1 mg*
Fluoxetine	yes	yes	oral solution 20 mg/5 mL (2002) *tablet 20 mg* *capsule 20, 60 mg*	yes	yes	oral solution 20 mg/5 mL (1991) *tablet 10–60 mg* *capsule 10–40 mg*
Folic acid	yes	yes	oral solution 2.5 mg/mL (2010) *tablet 0.4, 5 mg*	yes	no	*tablet 1 mg*
Furosemide	yes	yes	oral solution 20 mg/5 mL and 40 mg/5 mL (1998) *tablet 20, 40 mg*	yes	yes	oral solution 10 mg/mL, 40 mg/5 mL (1987) *tablet 20–80 mg*
Griseofulvine	yes	no	*tablet 125, 250, 500 mg*	yes	yes	oral suspension 125 mg/5 mL (2005) *tablet 250, 500 mg*
Haloperidol	yes	yes	oral solution 2 mg/mL, 1 mg/mL (1994)*tablet 10 mg*	yes	yes	oral solution 2 mg/mL (1993)*tablet 0.5–10 mg*
Hydrochlorothiazide	yes	no	*tablet 25, 50 mg*	yes	no	*tablet 12.5* *–50 mg* *capsule 12.5 mg*
Hydrocortisone	yes	yes	granule formulation 0.5, 1, 2, 5 mg (2018) *tablet 5–20 mg*	yes	no	*tablet 2.5* *–20 mg*
Hydroxicarbamide	yes	no	*capsule 200* *–500 mg*	yes	no	*capsule 100* *–1000 mg*
Hydroxychloroquine	yes	no	*tablet 200 mg*	yes	no	*tablet 200 mg*
Ibuprofen	yes	yes	oral suspension 20 mg/mL, 40 mg/mL (2004) powder for oral suspension 200–600 mg (2000) *tablet 200–600 mg*	yes	yes	oral suspension 100 mg/5 mL (1995) chewable tablet 50, 100 mg (1998) *tablet 400-800 mg*
Imatinib	yes	no	*tablet 50, 100, 400 mg* *capsule 100, 400 mg*	yes	no	*tablet 100, 400 mg*
Isoniazid	yes	no	*tablet 50, 100 mg*	yes	yes	syrup 50 mg/5 mL (1983) *tablet 100, 3000 mg*
Itraconazole	yes	yes	oral solution 10 mg/mL (1996) *capsule 100 mg*	yes	yes	oral solution 10 mg/mL (1997) *capsule 100 mg*
Ivermectin	yes	no	*tablet 3 mg*	yes	no	*tablet 3 mg*
Lactulose	yes	yes	oral solution 10 g/15 mL (1992)	yes	yes	oral solution 10g/15 mL (1992)
Lamivudine	yes	yes	oral solution 5 mg/mL and 10 mg/mL (1996) *tablet 150, 300 mg*	yes	yes	oral solution 5 mg/mL and 10 mg/mL (1995) *tablet 150, 300m g*
Lamotrigine	yes	yes	chewable tablet 2, 5, 25 mg (1994) *tablet 200 mg*	yes	yes	chewable tablet 2, 5, 25 mg (1998) *tablet 200 mg*
Levamisole	yes	no	*tablet 50 mg*	no	no	-
Levofloxacine	yes	no	*tablet 250, 500 mg*	yes	yes	oral solution 250 mg/10 mL (2011) *tablet 250, 500 mg*
Levothyroxin	yes	yes	oral solution 25 mcg/5 mL (2012) *tablet 25-200 mcg*	yes	no	*capsule 13* *–200 mcg*
Linezolid	yes	yes	oral solution 100 mg/5 mL (2001) *tablet 600 mg*	yes	yes	oral solution 100 mg/5 mL (2000) *tablet 600 mg*
Lopinavir	yes	yes	oral solution 80 mg/5 mL (2001) *tablet 100, 200 mg*	yes	yes	oral solution 80 mg/5 mL (2000) *tablet 100, 200 mg*
Loratadine	yes	yes	syrup 1 mg/mL (2001) *tablet 10 mg*	no	no	-
Mebendazole	yes	yes	oral suspension 20 mg/mL (1976) *tablet 100 mg*	yes	no	*tablet 100 mg*
Mefloquine	yes	no	*tablet 250 mg*	yes	no	*tablet 250 mg*
Mercaptopurine	yes	yes	oral suspension 20 mg/mL (2012) *tablet 50 mg*	yes	yes	oral suspension 50 mg/mL (2014) *tablet 50 mg*
Mesna	yes	no	*tablet 200, 400 mg*	yes	no	*tablet 400 mg*
Metformin	yes	yes	oral solution 500 mg/5 mL (2013) powder for oral suspension 850 mg (2013) *tablet 500–1000 mg*	yes	yes	oral solution 500 mg/5 mL (2003) *tablet 500–1000 mg*
Methadone	yes	yes	oral solution 1 mg/mL, 5 mg/mL (2002) oral concentrate 20 mg/mL (1996) *tablet 5–40mg*	yes	yes	oral solution 5 mg/5 mL, 10 mg/5 mL (1982) oral concentrate 20 mg/mL (1994) *tablet 5–40 mg*
Methotrexate	yes	yes	oral solution 2 mg/mL (2017) *tablet 2.5–10 mg*	yes	no	*tablet 2.5* *–15 mg*
Methylprednisolone	yes	no	*tablet 2* *–40 mg*	yes	no	*tablet 2* *–32 mg*
Metoclopramide	yes	yes	oral solution 1 mg/mL (1964) *tablet 5, 10 mg*	yes	yes	oral solution 1 mg/mL (1993) *tablet 5, 10 mg*
Metronidazole	yes	yes	oral suspension 125 mg/5 mL, 200 mg/5 mL (1969) *tablet 20–500 mg*	yes	no	*tablet 250, 500, 750 mg* *capsule 375 mg*
Midazolam	yes	yes	oromucosal solution in syringe 5 mg/mL (2012)	yes	yes	syrup 2 mg/mL (2002)
Miltefosine	no	no	-	yes	no	*capsule 50 mg*
Morphine	yes	yes	oral solution (multiple doses) (2003) *tablet (multiple doses)* *tablet extended release 5–200 mg*	yes	yes	oral solution (multiple doses) (2008) *tablet (multiple doses)* *tablet extended release 15–200 mg*
Moxifloxacin	yes	no	*tablet 400 mg*	yes	no	*tablet 400 mg*
Neostigmine	yes	no	*tablet 15 mg*	no	no	-
Nevirapine	yes	yes	oral suspension 50 mg/5 mL (2000) *tablet 200 mg*	yes	yes	oral suspension 50 mg/5 mL (1998) *tablet 200 mg*
Niclosamide	yes	no	*chewable tablet 500 mg*	no	no	-
Nifurtimox	no	no	*-*	no	no	-
Nilotinib	yes	no	*capsule 50, 150, 200 mg*	yes	no	*capsule 50, 150, 200 mg*
Nitrofurantoin	yes	yes	oral suspension 25 mg/5 mL (1960) *tablet 50–150 mg* *capsule 50–100 mg*	yes	yes	oral suspension 25 mg/5 mL (before 1982) *tablet 50–150 mg* *capsule 25–100 mg*
Nystatin	yes	yes	oral suspension 100,000 UI/mL (1957)	yes	yes	oral suspension 100,000 UI/mL (1983) *tablet 500,000 UI*
Omeprazole	yes	no	*capsule 10, 20, 40 mg*	yes	yes	powder for oral suspension 2.5, 10 mg (2008) *capsule 10, 20, 40 mg*
Ondansetron	yes	yes	oral solution 4 mg/5 mL (2014) syrup 4 mg/5 mL (2011) oral soluble film 4, 8 mg (2010) *tablet 4, 8 mg*	yes	yes	oral solution 4 mg/5 mL (1997) oral soluble film 4, 8 mg (1999) *tablet 4–24 mg*
Oseltamivir	yes	yes	oral suspension 6 mg/mL (2012) *capsule 30–75 mg*	yes	yes	oral suspension 6 mg/mL (2011) *capsule 30–75 mg*
Oxamniquine	no	no	-	yes	no	capsule 250 mg
P-aminosalicylic acid	no	no	-	yes	yes	powder for oral suspension 4 g (1982) *tablet 500, 1000 mg*
Paracetamol (acetaminophen)	yes	yes	oral solution 100 mg/mL (1971) *tablet 325–1000 mg*	yes	yes	*tablet 250, 650 mg*
Phenobarbital	yes	no	*tablet 15* *–100 mg*	no	no	-
Phenoxymethylpenicillin potassium	yes	yes	oral solution 250 mg/5 mL, 125 mg/5 mL (2012) powder for oral solution 250 mg (1987) *capsule 400 mg*	yes	yes	oral solution 250 mg/5 mL, 125 mg/5 mL (before 1982) *tablet 250, 500 mg*
Phenytoin sodium	yes	yes	oral suspension 125 mg/5 mL (1992) *tablet 100 mg*	yes	yes	oral suspension 125 mg/5 mL (before 1982) *chewable tablet 50 mg*
Phytomenadione	yes	yes	oral solution 2 mg/0.2 mL, 10 mg/1 mL (drinkable ampoule) (1957)	yes	yes	oral solution 10 mg/1 mL (drinkable ampoule) (1983)
Potassium iodide	yes	no	*tablet 100* *–300 μg*	no	no	-
Praziquantel	no	no	*-*	yes	no	*tablet 600 mg*
Prednisolone	yes	yes	oral suspension 10 mg/mL, 13.33 mg/mL (1969) *tablet 5–15 mg*	yes	yes	oral solution 5–20 mg/5 mL (1986) *tablet 10–30 mg*
Primaquine	no	no	*-*	yes	no	*tablet 15 mg*
Proguanil	yes	no	*tablet 100 mg (in association with atovacuone)*	yes	no	*tablet 100 mg (in association with atovacuone)*
Propranolol	yes	yes	oral solution 10 mg/5 mL, 40 mg/5 mL (2000) oral solution 3.75 mg/mL (2014) *tablet 10–80 mg*	yes	yes	oral solution 20 mg/5 mL, 40 mg/5 mL (1987) oral solution 4 mg/mL (2014) *tablet 10–80 mg*
Propylthiouracil	yes	no	*tablet 50 mg*	yes	no	*tablet 50 mg*
Pyrantel	yes	yes	oral suspension 250 mg/5 mL (1972) *chewable tablet 250 mg*	no	no	*-*
Pyrazinamide	yes	no	*tablet 250, 500 mg*	yes	no	*tablet 500 mg*
Pyridostigmine	yes	yes	syrup 12 mg/mL (1982) *tablet 60 mg*	yes	yes	syrup 60 mg/5 mL (before 1982) *tablet 60 mg*
Pyridoxine	yes	yes	oral solution 30.7 mg/mL (1969) *tablet 50, 300 mg*	no	no	-
Pyrimethamine	yes	no	*tablet 25 mg*	yes	no	*tablet 25 mg*
*Pyronaridine tetraphosphate*	no	no	*-*	no	no	*-*
Quinine	yes	no	*tablet 200 mg*	yes	no	*capsule 324 mg*
Raltegravir	yes	yes	powder for oral suspension 100 mg (2007) chewable tablet 25, 100 mg (2013) *tablet 400, 600 mg*	yes	yes	powder for oral suspension 100 mg (2013) chewable tablet 25, 100 mg (2011) *tablet 400, 600 mg*
Ranitidine	yes	yes	oral solution 150mg/10mL (2007) *tablet 150, 300 mg*	yes	yes	syrup 15 mg/mL (2007) *tablet 150, 300 mg* *capsule 150, 300 mg*
*Retinol*	no (available in association)	no	-	no	no	-
Ribavirin	yes	yes	oral solution 40 mg/mL (2005) *capsule 200 mg*	yes	yes	oral solution 40 mg/mL (2003) *tablet 200-600 mg* *capsule 200 mg*
*Riboflavin*	no (available in association)	no	-	no (available in association)	no	-
Rifampicin	yes	yes	oral suspension 100 mg/mL (1968) syrup 100 mg/5 mL *capsule 300 mg*	yes	no	*capsule 150, 300 mg*
Rifapentine	no	no	-	yes	no	*tablet 150 mg*
Ritonavir	yes	yes	oral solution 100 mg/mL (1996) *tablet 100 mg*	yes	yes	oral solution 100 mg/mL (1996) *tablet 100 mg*
Spironolactone	yes	no	*tablet 25, 50, 100 mg*	yes	no	*tablet 25, 50, 100 mg*
Stavudine	no	no	-	yes	yes	oral solution 1 mg/mL (1996) *capsule 15–40 mg*
Succimer	no	no	*-*	yes	no	*capsule 100 mg*
Sulfadiazine	yes	no	*tablet 500 mg*	yes	no	*tablet 500 mg*
Sulfametoxazole	yes	yes	oral suspension 40 mg/mL (1968) *tablet 100 mg*	yes	yes	oral suspension 200 mg/5 mL (1983) *tablet 400, 800 mg*
Thiamine	yes	no	*tablet 50, 100, 300 mg*	no	no	-
Tioguanine	yes	no	*tablet 40 mg*	yes	no	*tablet 40 mg*
Triclabendazole	no	no	-	no	no	-
Trimethoprim	yes	yes	oral suspension 8 mg/mL (1974) *tablet 20 mg*	yes	yes	oral solution 40 mg/5 mL (2000) *tablet 80, 160 mg*
Valganciclovir	yes	yes	oral solution 50 mg/mL (2008) *tablet 450 mg*	yes	yes	oral solution 50 mg/mL (2009) *tablet 450 mg*
Valproic acid	yes	yes	prolonged release granules 500 mg (2006) oral solution 200 mg/mL (1970) *tablet 500 mg*	yes	yes	syrup 250 mg/5 mL (before 1982) *capsule 250 mg*
Voriconazole	yes	yes	oral suspension 200 mg/5 mL (2004) *tablet 50, 200 mg*	yes	yes	oral suspension 200 mg/5 mL (2003) *tablet 50, 200 mg*
Warfarin	yes	yes	oral suspension 1 mg/mL (2010) *tablet 1–10 mg*	yes	no	*tablet 1* *–10 mg*
Zidovudine	yes	yes	oral solution 50 mg/5 mL (1991) *capsule 100, 250, 300 mg*	yes	yes	syrup 50 mg/5 mL (1989) *tablet 300 mg, capsule 100 mg*
Zinc sulfate	yes	no	*effervescent tablet 45 mg* *capsule 25 mg*	no	no	-
